# Investigation of Hepatitis B Virus and Human Immunodeficiency Virus Transmission among Severely Mentally Ill Residents at a Long Term Care Facility

**DOI:** 10.1371/journal.pone.0043252

**Published:** 2012-08-22

**Authors:** Supriya Jasuja, Nicola D. Thompson, Philip J. Peters, Yury E. Khudyakov, Megan T. Patel, Purisima Linchangco, Hong T. Thai, William M. Switzer, Anupama Shankar, Walid Heneine, Dale J. Hu, Anne C. Moorman, Susan I. Gerber

**Affiliations:** 1 Cook County Department of Public Health, Oak Forest, Illinois, United States of America; 2 Division of Viral Hepatitis, National Center for HIV, Hepatitis, STD, and TB Prevention, Centers for Disease Control and Prevention, Atlanta, Georgia, United States of America; 3 Division of HIV/AIDS Prevention, National Center for HIV, Hepatitis, STD, and TB Prevention, Centers for Disease Control and Prevention, Atlanta, Georgia, United States of America; Asociacion Civil Impacta Salud y Educacion, Peru

## Abstract

**Background:**

A high prevalence of hepatitis B virus (HBV) and human immunodeficiency virus (HIV) infections have been reported among persons with severe mental illness. In October, 2009, the Cook County Department of Public Health (CCDPH) initiated an investigation following notification of a cluster of HBV infections among mentally ill residents at a long term care facility (LTCF).

**Methods:**

LTCF staff were interviewed and resident medical records were reviewed. Residents were offered testing for HBV, HCV, and HIV. Serum specimens from residents diagnosed with HBV or HIV infection were sent to the Centers for Disease Control and Prevention (CDC) for analysis.

**Results:**

Eleven newly diagnosed HBV infections were identified among mentally ill residents at the LTCF. Of these 11 infections, 4 serum specimens were available for complete HBV genome sequencing; all 4 genomes were found to be closely related. Four newly diagnosed HIV infections were identified within this same population. Upon molecular analysis, 2 of 4 HIV sequences from these new infections were found to be nearly identical and formed a tight phylogenetic cluster.

**Conclusions:**

HBV and HIV transmission was identified among mentally ill residents of this LTCF. Continued efforts are needed to prevent bloodborne pathogen transmission among mentally ill residents in LTCFs.

## Introduction

A high prevalence of hepatitis B virus (HBV), hepatitis C virus (HCV), and human immunodeficiency virus (HIV) infections have been reported among persons with severe mental illness [1], [2]. In addition, several outbreaks of HBV infection have occurred among persons who reside in long term care facilities (LTCFs). Reported modes of transmission include health-care acquired (inappropriate re-use of blood-contaminated medical equipment such as fingerstick devices and podiatry instruments) and behavioral factors (intravenous drug use and high-risk sexual practices) [3], [4]. To our knowledge, outbreaks of HIV infection have not been reported in LTCFs in the United States, although HIV infection outbreaks have been documented in institutionalized [5] and health care settings [6]. In October 2009, four cases of acute HBV infection among residents with severe mental illness at LTCF A were reported to the Cook County Department of Public Health (CCDPH) and an investigation was initiated to evaluate for evidence of bloodborne pathogen transmission within LTCF A and to prevent further infections.

## Methods

### Setting

LTCF A was a three-story building that housed 280 residents. The facility included a chronic care unit with 180 psychiatric (primarily with diagnoses of schizophrenia and bipolar disease) and Alzheimer's residents. The remaining 100 residents were housed in a skilled nursing unit. During this investigation of LTCF A the average daily occupancy was 97% and the median length of stay was 200 days.

### Case finding and laboratory testing

LTCF A staff were interviewed and resident medical records were reviewed to evaluate for exposures that could be implicated in healthcare-associated bloodborne pathogen transmission and for other potential modes of transmission (e.g., high-risk sexual behavior). Outbreak investigations are reportable in Illinois. Outbreak investigations include review of medical charts and interviews of patients and healthcare providers. HBV, HCV, and HIV infections were defined based on medical chart documentation and by screening tests offered to all residents. All residents were offered testing for HBV, HCV, and HIV in October, 2009 and again after 3 and 6 months. Hepatitis B surface antigen (HBsAg) and hepatitis B surface antibody (anti-HBs) was detected by the VITROS® HBsAg assay and Anti-HBs assay (Ortho Clinical Diagnostics, Raritan, New Jersey). HBsAg positive specimens were then tested for IgM antibody to hepatitis B core antigen by the Advair Centaur ® anti-HBc IgM (Siemens Healthcare Diagnostics, Tarrytown, NY). Acute HBV infection was defined by a positive HBsAg and positive anti-HBc IgM. Chronic HBV infection was defined by a positive HBsAg and a negative anti-HBc IgM. HCV screening was performed with the VITROS® Anti-HCV assay (Ortho Clinical Diagnostics, Raritan, New Jersey). HIV testing was performed with the Clearview HIV-1/2 Stat-Pack (Inverness Diagnostics, Princeton, New Jersey) and reactive results were confirmed by Bio-Rad GS™ HIV-1 Western Blot Kit (Bio-Rad Laboratories, Redmond, Washington).

### Molecular testing of HBV and HIV viruses

Residual serum specimens (when available) from residents diagnosed with acute and chronic HBV infection were sent to the Division of Viral Hepatitis at the Centers for Disease Control and Prevention (CDC). HBV DNA viral load was quantified with the COBAS Amplicor Monitor v2.0 (Roche Molecular Diagnostics, Pleasanton, California) and HBV genotypes were determined with the INNO-LiPA HBV Genotyping Assay (Innogenetics N.V., Ghent, Belgium). Detectable HBV DNA specimens were used to identify full genome sequences of HBV variants and to compare these sequences with each other and representative reference sequences using phylogenetic analysis as described [7].

Residents diagnosed with HIV infection who had been tested for evidence of HIV antiretroviral resistance with *pol* gene sequencing (HIV GenoSure), had *pol* sequences compared with each other and with representative reference sequences using phylogenetic analysis at CDC [8]. The sequences were also evaluated with a genotypic resistance interpretation algorithm (http://sierra2.stanford.edu/sierra/servlet/JSierra) which gives inferred levels of resistance to common antiretrovirals.

## Results

### Epidemiologic Investigation

During March–October 2009, HBV infection was clinically diagnosed in seven residents. Upon recognition of the outbreak, screening for HBV, HCV, and HIV was offered to all residents. No newly diagnosed infections of HBV, HCV, or HIV were identified among the residents of the skilled nursing facility. Among the approximately 180 residents of the chronic care unit, a majority consented to HBV (n = 171), HCV (n = 165), and HIV (n = 143) testing. HBV infection was detected in four additional residents during the initial screening. Of the total 11 newly diagnosed HBV infections, 8 were in the acute phase and 3 were in the chronic phase. The median age of the residents with HBV infection was 47 years (range 34–62 years) and 78% were male. All of the HBV infections occurred among residents with severe mental illness and these residents had resided at LTCF A for a median of 25 months (range 0.3–61 months). No additional HBV infections were identified among residents of the chronic care unit at the 3 and 6 month follow-up testing.

Among 165 residents of the chronic care unit who were screened for HCV, 36 (22%) had a positive HCV serology result. All 36 residents with a positive HCV serology result had severe mental illness and 28 (78%) had not previously been reported to the Illinois viral hepatitis registry. Residents with a positive HCV serology result were referred for confirmatory testing and medical care.

Seven residents were known to have HIV infection prior to the investigation period. Four additional residents were newly diagnosed with HIV infection during this time period including one identified early in the outbreak, two diagnosed during the initial HIV screening, and one diagnosed at the 3 month follow-up screening. These residents with newly diagnosed HIV infection had resided at LTCF A for a median of 15 months (range 8–26 months) and two of the four also had newly diagnosed chronic HBV co-infection.

Review of resident medical charts did not reveal an association between specific healthcare provider visits or medical procedures and HBV or HIV infection. Facility staff interviews revealed that residents were sexually active, however, information on specific partners and frequency of encounters was not available. The majority of mentally ill residents were ambulatory and could leave LCTF A for unmonitored visits to the community. Interviews conducted with residents were limited because of severity of mental illness.

### Molecular and Phylogenetic Analysis

**Figure 1:**
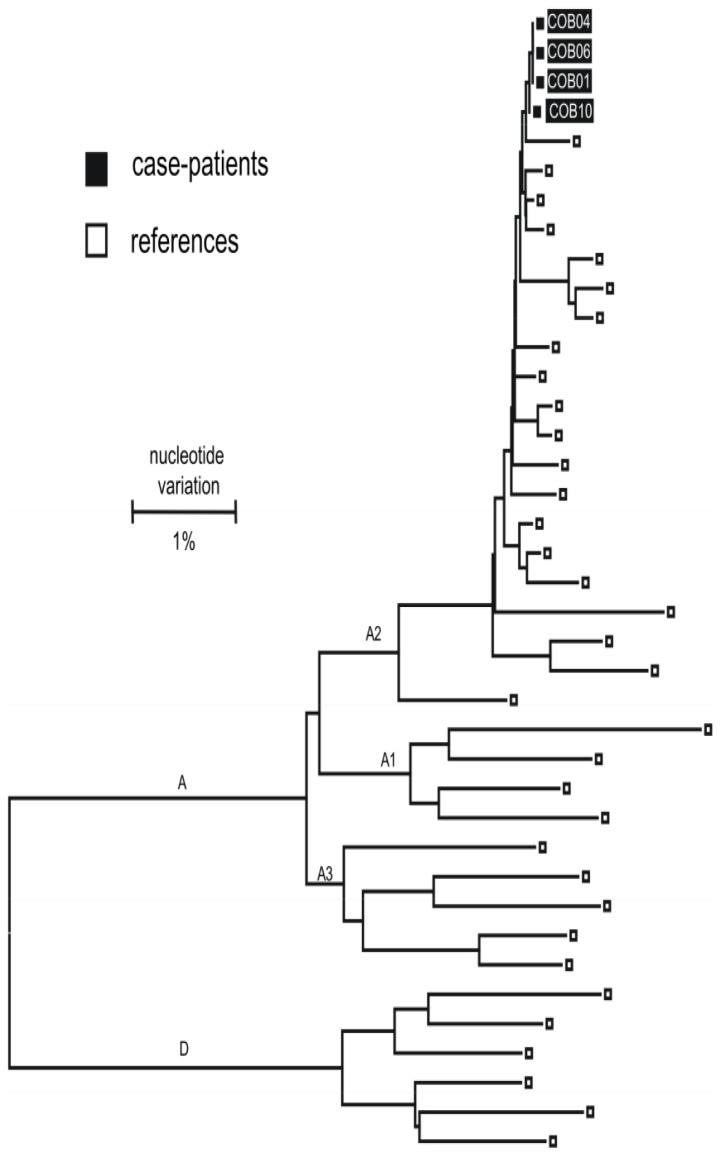
Phylogenetic relationship of the complete HBV genomic sequences of 4 long term care facility (LTCF) A residents with newly diagnosed HBV infection and representative HBV genotype A (n = 29) and D (n = 6) strains. Representative HBV genotype A and D strains were retrieved from GenBank and CDC's sequence database. The 4 LTCF A residents are shown by the solid boxes.

From the 11 residents newly diagnosed with acute or chronic HBV infection, 7 serum specimens (5 acute and 2 chronic) were sent to CDC for HBV DNA sequence analysis. HBV DNA was detected in the sera from 6 of 7 residents (viral load range: 126–2,179,179,000 IU/mL). The S gene region (441 base pairs in length) was sequenced in these 6 HBV DNA positive specimens. All 6 were HBV sub-genotype A2 and had identical S-gene sequences. Complete HBV genome sequences were obtained from four of these specimens (2 acute and 2 chronic) and all 4 sequences were closely related (nucleotide identities 99.97% to 100%) and formed a single cluster [Figure 1]. There were no HBV drug resistance mutations detected.

**Figure 2:**
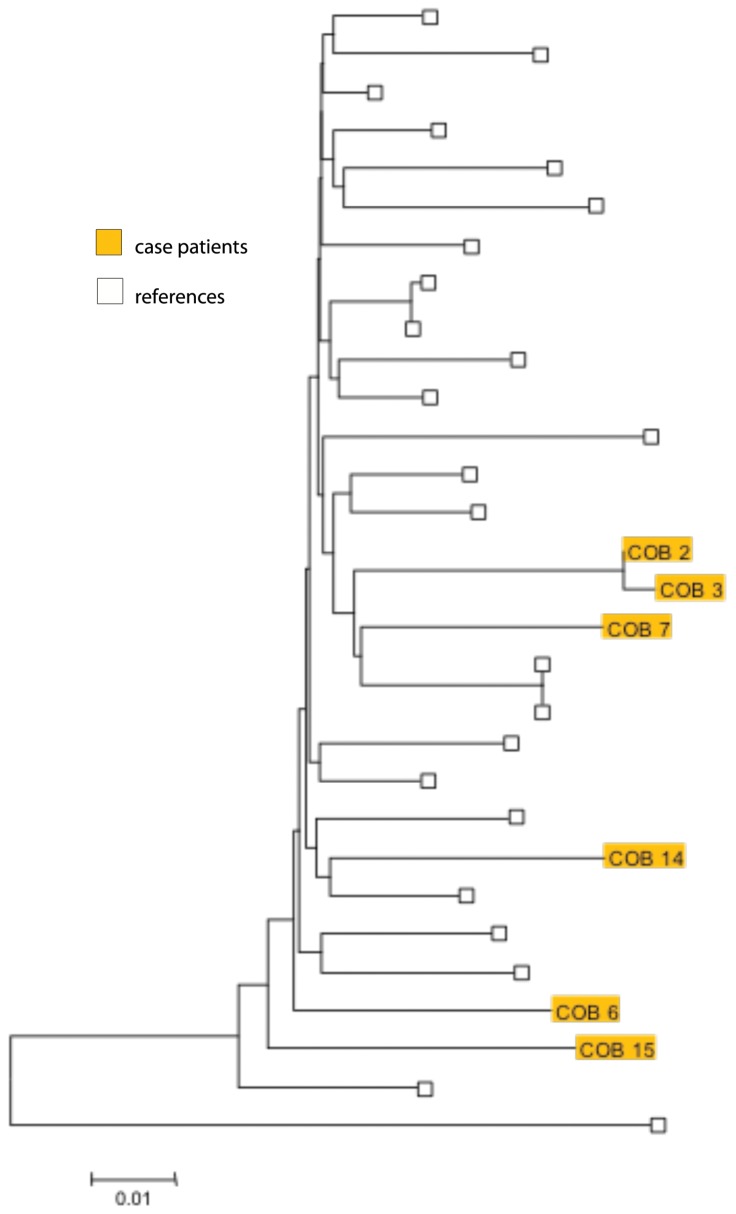
Phylogenetic relationship of the HIV sequences of 6 LTCF A residents with HIV infection (4 newly diagnosed), 23 reference US subtype B sequences, and one reference subtype C sequence (the outgroup). The 6 LTCF A residents are shown by the solid boxes. The tree was derived from a nucleotide alignment of the 1497-bp *pro-pol* region. Trees were inferred from 3 different phylogenetic analysis methods (neighbor-joining, maximum likelihood, and Bayesian inference) which independently showed that 2 sequences (COB 2 and COB 3) consistently cluster together with high bootstrap/statistical support (99/100/1.0 respectively). These two sequences show a pairwise nucleotide identity of 99.3%.

HIV *pol* gene sequences were available for 6 (including all 4 new diagnoses and 2 previously known HIV-infected residents) of the 11 HIV-infected residents. Two sequences (both new diagnoses) clustered together with high bootstrapping and statistical support (shared pairwise nucleotide identity of 99.3%). Although none of the newly diagnosed residents had been previously exposed to antiretroviral medications, 3 of 4 had drug-resistant viruses. The two newly diagnosed sequences that clustered also shared the same drug resistance mutations (T69N, K219K/Q, G190A and K238T). The third newly diagnosed sequence with drug resistance mutations had K70R, M184V and L90M. [Figure 2]

## Discussion

This outbreak investigation identified evidence of both HBV and HIV transmission among residents with severe mental illness at a LTCF. Although a high prevalence of HBV and HIV infections has been documented among severely mentally ill persons [1], our investigation suggests this population remains at risk for newly acquiring these infections even while receiving care within a LTCF setting. Although this investigation was not able to demonstrate the mode of HBV and HIV transmission, it highlighted several strategies (such as routine screening for bloodborne pathogens [9], HBV vaccination, access to condoms, and risk-reduction education) which could prevent the spread of these infections among mentally ill residents of LTCFs.

Overall 8 residents were diagnosed with acute HBV infection and 3 residents were diagnosed with chronic HBV infection. Although these residents were diagnosed at different phases of their infection (acute vs. chronic), these infections may have all been acquired during their stay in the facility. This is supported by the finding that viruses obtained from 2 residents with chronic HBV infections were closely related to viruses obtained from 4 residents with acute HBV infections. This suggests that all residents with available complete HBV genome sequences likely belonged to a single transmission network. One of the residents with chronic HBV infection may have served as a reservoir for HBV transmission within LTCF A. Alternatively the initial source of HBV infection may have been an individual who had left LTCF A prior to the initiation of the investigation and therefore was never tested.

Among the 4 new HIV diagnoses, *pol* gene sequencing revealed that 2 sequences clustered and that 3 sequences had evidence of transmitted drug resistance. Although clustering does not establish an epidemiologic link between cases (intermediaries may exist), it does suggest that they are part of the same transmission network and interrupting transmission in these networks may be particularly important given the high proportion with transmitted drug resistance. Although integrating HIV medical care with psychiatric and addictive disorders care has been proposed [10], reducing HIV transmission among individuals with mental illness in LTCFs will also require integrating HIV prevention strategies that are tailored to these populations and their drug use and sexual networks.

Previous studies have shown that persons with severe mental illness engage frequently in high-risk behaviors including reported intravenous drug use [11] and risky sexual behavior [1], [2], [12]. The mentally ill residents of this LTCF were ambulatory and had unmonitored visits outside the facility. In addition, sexual activity among these residents was frequent according to staff interviews. Because of the potential for multiple sex partners and illicit intravenous drugs in the community, there may have been multiple routes of transmission of bloodborne pathogens among this cohort of mentally ill residents. Preventive interventions that target substance abuse and promote condom use during sex remain important tools to prevent new HBV, HCV, and HIV infections in mentally ill persons residing in LTCFs [13]. While there are longstanding recommendations for routine hepatitis B vaccination for developmentally disabled persons in congregate living settings [14], no similar guidelines exist that recommend HBV vaccination among mentally ill residents in congregate housing. Our findings suggest a need for hepatitis B vaccination guidelines to be extended to include mentally ill residents in congregate settings.

Several limitations were encountered during this investigation. First, it was not possible to perform standardized interviews with this patient population because the severity of the mental illnesses of these residents precluded accurate answers with respect to risk factors for transmission of blood-borne pathogens. Second, it was not possible to track the whereabouts of community visits for the patients who were allowed to leave the facility. Third, medical records were limited with respect to documentation and previous results of HBV and HIV testing. These limitations led us to evaluate for molecular evidence of transmission and these molecular phylogenetic analyses provided strong additional evidence of HBV and HIV transmission among residents of this facility.

CCDPH made several recommendations to LTCF A to control and prevent further transmission among their residents. LTCF A was advised to screen mentally ill patients for HIV, HBV, and HCV at time of initiation of the outbreak investigation and then again at 3 and 6 months in order to identify additional infections. Ninety out of 160 susceptible residents who were eligible for HBV vaccination received the vaccine at least once. Resident education regarding safe sex practices was reinforced. There were no further acute HBV infections identified in the 6 months after the initiation of the investigation.

This outbreak demonstrates the need for continued efforts to prevent bloodborne pathogen transmission among severely mentally ill residents who reside in LTCFs. Screening for bloodborne pathogens [9] including HIV, HBV, and HCV at the time of admission to a LTCF and at regular intervals thereafter is an important strategy to identify infected residents. HBV vaccination policies such as those recommended for mentally disabled individuals in congregate settings, should be considered for severely mentally ill in these types of LTCFs. Lastly, strategies aimed at risk reduction will be important to prevent further spread of these infections among LTCF populations.
